# Crop Diversity for Yield Increase

**DOI:** 10.1371/journal.pone.0008049

**Published:** 2009-11-26

**Authors:** Chengyun Li, Xiahong He, Shusheng Zhu, Huiping Zhou, Yunyue Wang, Yan Li, Jing Yang, Jinxiang Fan, Jincheng Yang, Guibin Wang, Yunfu Long, Jiayou Xu, Yongsheng Tang, Gaohui Zhao, Jianrong Yang, Lin Liu, Yan Sun, Yong Xie, Haining Wang, Youyong Zhu

**Affiliations:** 1 Key Laboratory of Agro-Biodiversity and Pest Management of Education Ministry of China, Yunnan Agricultural University, Kunming, Yunnan, China; 2 Plant Protection Station of Honghe Prefecture, Mengzi, Yunnan, China; 3 Agroscience Research Institute of Yuxi City, Yuxi, Yunnan, China; 4 Plant Protection Station of Chuxiong Prefecture, Chuxiong, Yunnan, China; 5 Plant Protection Station of Shiping County, Shiping, Yunnan, China; 6 Agroscience Research Station of Hongxi Town, Mile, Yunnan, China; 7 Agricultural Technology Extension Centre of Qujing City, Qujing, Yunnan, China; 8 Agricultural Technology Extension Centre of Zhaotong City, Zhaotong, Yunnan, China; 9 Agricultural Technology Extension Centre of Lincang City, Lincang, Yunnan, China; University College London, United Kingdom

## Abstract

Traditional farming practices suggest that cultivation of a mixture of crop species in the same field through temporal and spatial management may be advantageous in boosting yields and preventing disease, but evidence from large-scale field testing is limited. Increasing crop diversity through intercropping addresses the problem of increasing land utilization and crop productivity. In collaboration with farmers and extension personnel, we tested intercropping of tobacco, maize, sugarcane, potato, wheat and broad bean – either by relay cropping or by mixing crop species based on differences in their heights, and practiced these patterns on 15,302 hectares in ten counties in Yunnan Province, China. The results of observation plots within these areas showed that some combinations increased crop yields for the same season between 33.2 and 84.7% and reached a land equivalent ratio (LER) of between 1.31 and 1.84. This approach can be easily applied in developing countries, which is crucial in face of dwindling arable land and increasing food demand.

## Introduction

It has been recognized that biodiversity is key to securing global food supply[1]. Zhu *et al.* reported that planting a mixture of rice varieties was effective in boosting yields[2] and decreasing diseases[3]. The approach had been extended to 1.57million ha from 2000 to 2004 in 11 provinces of China. It increased 675 kg/ha yield in average and 259 million US$ of income and cost-saving. Rice blast in mixtures was 67% less severe than that in monoculture[4]. In natural ecological systems, it has been shown that biomass production can be elevated with increasing biodiversity[5], [6]. For example, Tilman *et al.* showed that biomass production from experimental fields in which 16 grass species were grown in a mixture was increased by 2.7 times compared with those in which single species were grown alone[7]. They also demonstrated that the more plant species a field contained the more stable the ecological system was from year to year[8]. This accords with observations by Li *et al*.[9], Morgado and Willey[10] and Dybzinski *et al.* [11] that biodiversity could increase soil fertility and LER. In crop systems, there is great potential for the use of mixed cropping to enhance productivity, but this must be tested at a scale relevant to agricultural production[12]–[13].

## Results

**Figure 1:**
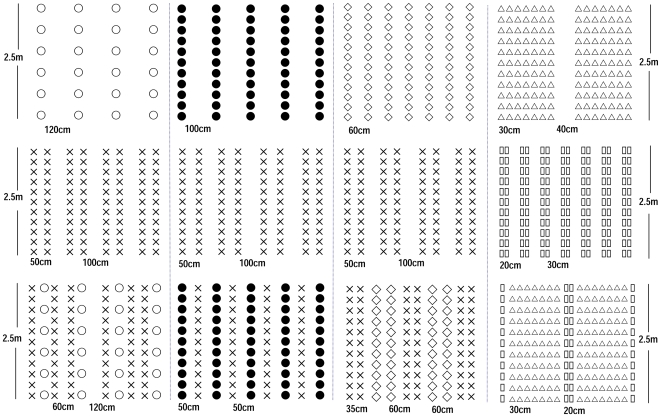
Crop patterns in intercropping and monoculture experiment plots. Each symbol represents a plant (hill) of a different crop species: tobacco (○); maize (×); sugarcane (•); potato (⋄); wheat (▵); broad bean (□).

In collaboration with farmers and extension personnel in 10 counties in Yunnan Province, we tested intercropping of tobacco-maize, sugarcane-maize, potato-maize, and wheat-broad bean, either by overlapping growing seasons or by mixing crop species based on differences in their heights. The four crop combinations were compared with their respective monocrops in adjacent plots. A schematic illustration of the planting arrangements is shown in Figure 1 with details of the planting and harvesting dates.

Yunnan is the key plantation region for tobacco in China, with a cultivation area of over 400,000 ha. Local farmers grow tobacco in summer and wheat or barley in winter; tobacco is normally harvested in mid-August and planting of wheat or barley does not begin until November, leaving the fields unutilized for three months. By planting maize in the tobacco field in mid-July and harvesting in November, an additional crop can be grown in this period.

**Figure 2:**
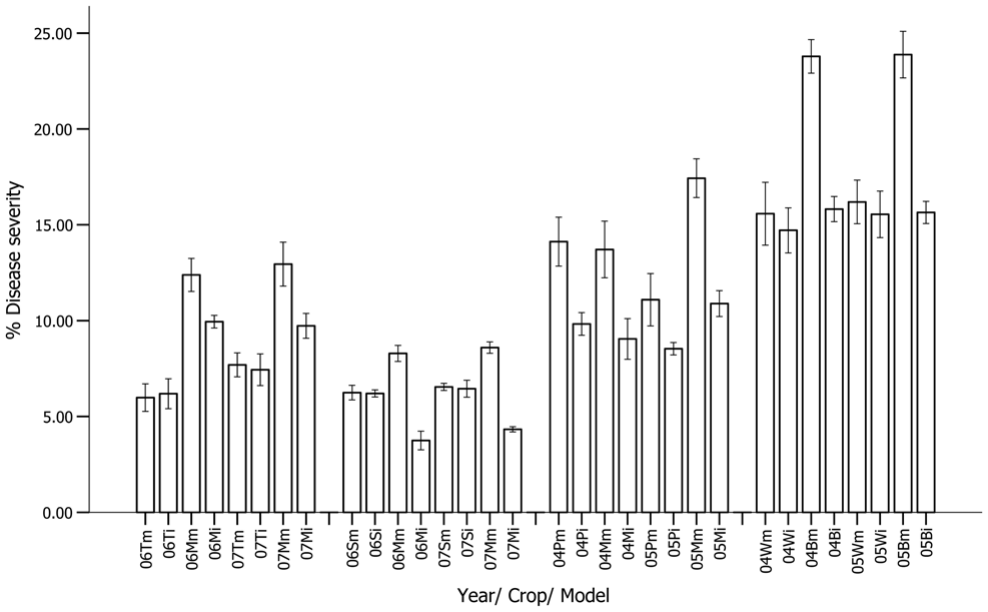
Severity of main diseases of the crops in monocropping and intercropping systems. T = Tobacco brown leaf spot (*Alternaria alternate* Keissler); M = Maize northern leaf blight (*Setosphaeria turcica* Leonard); S = Sugarcane eye spot (*Bipolaris sacchari* (Butl) Shoemaker); P = Potato late blight (*Phytophthora infestans* (Mont.) de Bary); W = Wheat Stripe Rust (*Puccinia striiformis* West); B = Broad bean *chocolate* spot (*Botrytis fabae* Sard). m = disease severity for crop species grown in monoculture control plots; i = disease severity for the same crop species grown in intercropping plots in the same fields. Error bars are one s. e. m; n = 3. Statistical analyses were conducted by software SPSS 13.0. All differences between pairs are significant at P≤0.05 based on one-tailed t-test.

We tested intercropping of tobacco-maize in Mile, Yao'an and Chuxiong counties, and this pattern was adopted by local farmers in 325 ha and 4,162 ha of farmland in 2006 and 2007, respectively. The results show that the yields of tobacco were comparable in both systems. Intercropping resulted in additional maize production of 5.88 and 5.91 t/ha in 2006 and 2007, respectively (Table 1), constituting 84.7 and 84.5% of the production from the monocrops, with LERs of 1.84 and 1.83. Severity of tobacco brown leaf spot disease in the two systems was comparable, but northern maize leaf blight in intercropped plots was decreased by 17.0 and 19.7% in 2006 and 2007, respectively, compared with the monocropped controls (Fig. 2).

**Table 1:** Yield and monetary value for different crops.

Crop	Variety	Plants m^−2^	Yield ± s. e. m (t/ha)		Crop value (US$ per ha)	
			1st year	2nd year	1st year	2nd year
Tobacco	Yunyan-87	1.67	2.82±0.003	2.86±0.007	5829	5912
Maize	Huidan-4	5.35	6.94±0.003	6.99±0.017	1972	1986
Intercropping		6.67	8.69	8.75	7477	7477
Tobacco	Yunyan-87	1.67	2.81±0.006	2.84±0.017	5808	5870
Maize	Huidan-4	5.00	5.88±0.004	5.91±0.017	1671	1679
Sugarcane	Xintaitan-2	9.62	105.87±0.851	105.23±0.256	2529	2514
Maize	Xundan-7	5.35	7.54±0.006	7.47±0.030	2142	2123
Intercropping		13.45	110.35	111.67	3878	3878
Sugarcane	Xintaitan-2	9.45	105.58±0.575	106.95±0.409	2522	2555
Maize	Xundan-7	4.00	4.77±0.005	4.72±0.020	1355	1341
Potato	Hui-2	6.67	31.86±0.105	31.27±0.380	2058	2020
Maize	Huidan-4	5.35	7.17±0.022	7.13±0.026	2037	2026
Intercropping		7.42	23.71	23.99	2687	2687
Potato	Hui-2	3.71	18.45**(115)**	18.75**(120)**	1192	1211
Maize	Huidan-4	3.71	5.26**(147)**	5.24**(147)**	1495	1489
Wheat	Yumai-3	277.36	5.31±0.013	5.32±0.016	1577	1580
Broad bean	Dabaidou	13.65	2.87±0.011	2.92±0.011	1389	1413
Intercropping		280.05	6.27	6.28	2045	2045
Wheat	Yumai-3	277.36	5.29±0.020	5.31±0.017	1571	1577
Broad bean	Dabaidou	2.69	0.98±0.012	0.97±0.007	474	469

Crop yield determined by grain weight for rice, wheat and broad bean, dry leaf weight for tobacco, fresh stem and tuber weight for sugarcane and potato. Crop values based on market prices of 2067.02 US$ per ton for tobacco, 284.15 US$ per ton for maize, 23.89 US$ per ton for sugarcane, 64.59 US$ per ton for potato, 296.98 US$ per ton for wheat, 483.97 US$ per ton for broad bean. Crop yield and value were for individual species within intercropping. Yields of tobacco-maize, sugarcane-maize and wheat-broad bean patterns were additional production compared with monocrops. Yields of potato intercropped with maize and maize intercropped with potato, compared with equal areas of monocrops are shown in **(bold)**. Statistical analyses: each survey plot was considered to be an experimental unit, and analyses were based on actual mean plot yields. Statistical analyses were conducted by software SPSS 13.0. One-tailed t-tests were used to determine if the yield differed significantly (p≤0.05).

This approach was also applied to crops with long cultivation seasons. Sugarcane, with a cultivation area of about 300,000 ha in Yunnan, has a year-long cultivation season. As the plants are short in the first half of the season, there is sufficient light available to grow an additional crop of maize in this period. Sugarcane-maize was tested in Mile, Shiping and Yongde counties, and adopted by local farmers in 80 ha and 1,582 ha of farmland in 2006 and 2007 respectively.

The results show that the yields of sugarcane were comparable between monocropped and intercropped plots. The intercropped maize produced an additional 4.77 and 4.72 t/ha in 2006 and 2007, respectively (Table 1), constituting 64.0 and 63.2% of the production from the respective monocrops, with LERs of 1.63 and 1.64. Severity of sugarcane eye spot disease in the two systems was comparable, but northern maize leaf blight in intercropped plots decreased by 55.9 and 49.6% in 2006 and 2007, respectively, compared with the monocrops (Fig. 2). This reduction in maize disease may be a result of less rainfall during the earlier growing period of the intercropped plots.

We also intercropped short and tall crops in the same field, increasing the spatial utilization of farmland. By working with farmers in Xuanwei, Huize and Zhaotong counties, we intercropped potato-maize in 1,685 and 5,658 ha of farmland in 2004 and 2005, respectively. The maize yields from intercropping were 147% in both years compared with equal areas of the monocrops. The intercropped potato yields in these two years were 115 and 120% compared with equal areas of monocrops (Table 1), resulting in LERs of 1.31 and 1.33. Severity of potato late blight in the intercropping system was decreased by 32.9 and 39.4% in 2004 and 2005, respectively, compared to the monocrops, while northern maize leaf blight in intercropped plots was decreased by 30.4 and 23.1% (Fig. 2).

Similar experiments were also carried out with crops of similar cultivation seasons. In 2004 and 2005, we intercropped wheat and broad bean in 358 ha and 1,452 ha of farmland, respectively, in Hongta and Yimeng counties (Fig. 1). The results show that wheat yields from intercropping were comparable with the monocrops in both years. Intercropping resulted in additional broad bean production of 0.98 and 0.97 t/ha, in 2004 and 2005, respectively (Table 1), constituting 34.2 and 33.2% of the production from the monocrops, giving LERs of 1.34 and 1.33 in 2004 and 2005 (Table 2). Severity of broad bean chocolate spot disease in the intercrops decreased by 33.8 and 31.7% in 2004 and 2005, respectively, compared with the monocrops (Fig. 2). This could result from reduction in disease spread among broad bean plants because they were separated by rows of wheat plants.

**Table 2:** Land equivalent ratios for crop yields produced by intercropping.

Intercropping	First year	Second year
Tobacco/Maize	1.84	1.83
Sugarcane/Maize	1.63	1.65
Potato/Maize	1.31	1.33
Wheat/Broad bean	1.34	1.33

Land equivalent ratios (LERs) were calculated as (yield ha^−1^ of crop A in intercropping/yield ha^−1^ of crop A in monoculture)+(yield ha^−1^ of crop B in intercropping/yield ha^−1^ of crop B in monoculture).

## Discussion

These large-scale experiments demonstrate the advantages of cultivating a mixture of crop species in the same field through temporal and spatial management. Intercropping maize in tobacco and sugarcane enhanced utilization of land space and physical resources during the late growing period of tobacco and early cultivation phase of sugarcane, adding a season of maize production. In the conventional practice, only one crop was cultivated in the field during growing season. For the tested patterns in the study, all the practice for tobacco and sugarcane was exactly adopted as same as conventional practice. Maize was extra crop for tobacco and sugarcane fields. The combination of potatoes with maize as well as wheat with broad bean took advantage of the differences in their heights. Such intercropping resulted in the formation of three-dimensional crop assemblies in the fields, possibly improving growth through a more favorable microclimate. These systems boosted yields and reduced disease, produced high LERs and increased farmers' incomes (Table 1; Table 2; Fig. 2), although they required higher labors inputs, more seeds and fertilizer.

Intercropping short and tall plants may benefit crop growth by increasing light and air diffusion. The reduction in potato late blight disease in intercropped plots may be a result of less rainfall during the growing period between April and July compared with the monocrops between June and August, when the disease normally peaks[14]. After the potato crop was harvested, the ambient humidity and leaf wetness of the maize decreased because of the distance between the rows of plants, which may limit the spread of the northern leaf blight. Because of these beneficial effects, this intercropping design has been adopted by most of the local farmers.

Taken together, our large-scale intercropping experiments in 15,302 ha of farmland have provided an unprecedented amount of data that demonstrate intercropping's clear advantages of boosting yields and preventing disease. Our results support the findings on the relationship between biodiversity and biomass production based on perennial plant populations in experimental models[15]. Our studies involved farmers from the beginning, an approach that helped them to understand the rationale behind the technique and enabled rapid assimilation of research results among the local communities.

In addition to pointing to the importance of crop diversity, our findings have wide implications for food security. Increasing food production by intercropping is very simple and can be easily applied in developing countries, which is crucial in the face of dwindling arable land and increasing food demand. It has been recognized that reduction in arable land is one of the key factors in causing the current food crisis[16]. In China, the area of arable land was reduced by 4.7 Mha, or about 4.5% between 1978 and 1996[17]. A report by FAO projects that the area of arable land per person in the world might decrease below the critical level of 0.1 hectare by 2050 due to increasing desertification and urbanization[16]. As the reduction in arable land is unavoidable, increasing LER and food production per unit area is crucial for securing food supply. The crop diversity techniques described in this paper have been listed by the Yunnan Provincial Government as a key strategy to boost food production and is applied to 1.5 Mha per year, according to Provincial Government statistics. It is crucial that such a simple, effective approach to boosting crop yields and increasing LERs is widely adopted in the global challenge of securing the food supply.

## Materials and Methods

### Field Study

The field experiment sites of different crops combination were located in different areas which were suitable for these crops growth. The experiment sites were chosen in the most suitable cultivation area for different crop combinations. Tobacco-maize sites were located in Mile, Yao'an and Chuxiong Counties ranging from 1000 m to 1600 m (a.s.l.), Sugarcane-maize sites in Shiping, Mile and Yongde Counties under 1200 m (a.s.l), Maize-potato sites in Xuanwei, Huize and Zhaotong Counties ranging from 1600 m to 2100 m (a.s.l.), Wheat-broad bean sites in Hongta and Yimen Counties ranging from 1500 m to 1900 m (a.s.l.). Each crop combination was tested in three different experiment sites. Each site included three treatments, i.e. one treatment for intercropping, and the other two treatments for monoculture of the two crops (Fig. 1). Each treatment with three replicates (3×3), nine plots for each site were located in the same field by randomized blocks design.

Field surveys were carried out in 2004–2007, with experimental crop patterns adopted by farmers in Yunnan Province. There were three experimental plots (each about 200 m^2^) for each crop combination each year. Planting and harvest time for each crop combination were the same for each year and field management was conducted by farmers according to local practice. For tobacco-maize, tobacco seedlings (Yunyan-87) were planted on 22 April and harvested progressively between 15 June and 18 August. To intercrop, maize seeds (Huidan-2) were planted in the tobacco fields on 10 July and harvested on 30 October. For monocropping, tobacco was grown in the same way as the intercrop, whereas maize cultivation followed the usual timeframe – sown 25 May and harvested 18 September. For sugarcane-maize, sugarcanes (Xintaitang-2) were planted on 5 January and harvested on 25 December; maize seeds (Xundan-7) were planted in the sugarcane fields on 20 February and harvested on 30 June. For monocropping, sugarcane was grown in the same way as the intercrop, maize in the usual timeframe, as above. For potato-maize, the potato cultivation season was shifted seven weeks earlier: the Hui-2 variety was planted 30 March and harvested 15 July. Maize (Huidan-4) was grown in the usual timeframe, as above. For monocropping, cultivation of both potato and maize followed their usual timeframe, with potatoes planted 20 May and harvested 2 September, and with maize planted and harvested as described above. With wheat-broad bean, for both intercropping and monoculture, wheat (Yunmai-3) was sown 28 October and harvested 25 April; broad bean (Dabaidou) was planted 10 October and harvested 5 April.

### Yield and Monetary Value Surveys

The yield data in Table 1 were based on whole plots harvest. Crop yield was determined by grain weight for Maize, wheat and broad bean, dry leaf weight for tobacco, and fresh stem and tuber weight for sugarcane and potato. Crop values were based on market prices of 2067.02 US$ per ton for tobacco, 284.15 US$ per ton for maize, 23.89 US$ per ton for sugarcane, 64.59 US$ per ton for potato, 296.98 US$ per ton for wheat, and 483.97 US$ per ton for broad bean. Crop yield and value were for individual species within intercropping. Yields of tobacco-maize, sugarcane-maize and wheat-broad bean patterns were additional production compared with monocrops. Each survey plot was considered to be an experimental unit, and analyses were based on actual mean plot yields. Statistical analyses were conducted by software SPSS 13.0. One-tailed t-tests were used to determine if the yield differed significantly (p≤0.05).

### Severity of Crop Diseases

One of the most serious diseases for each crop was surveyed. Survey standard of tobacco brown leaf spot (*Alternaria alternate* Keissler) is based on the YC/T40-1996, P.R. China[18]; Maize northern leaf blight (*Setosphaeria turcica* Leonard) on the NY/T 1248.1-2006, P.R. China[19]; Wheat Stripe Rust (*Puccinia striiformis* West) on the NY/T 1443.1-2007, P.R. China[20]; Disease survey of sugarcane eye spot (*Bipolaris sacchari* (Butl) Shoemaker) followed Dai's report (1993) [21]; Potato late blight (*Phytophthora infestans* (Mont.) de Bary) followed Handbook of Crop Pest Forecasting (2006) [22]; Broad bean *chocolate* spot (*Botrytis fabae* Sard) followed Wallen's report (1957) [23].

All investigated diseases were assessed at five sampling points in each plot, distributed in a uniform pattern. Sampling number, sampling plant part and disease categories were different described as below.

Tobacco brown leaf spot (*Alternaria alternate* Keissler) disease: whole leaves of twenty plants were evaluated at each sampling point. Five disease scales were rated in terms of the percentage of symptomatic leaf area: 0, no disease; 0.5, less than 1%; 1, 1–5%; 2, 5–10%; 3, 10–20%, 4, >20% leaf area affected.

Maize northern leaf blight [*Setosphaeria turcica* (Pass.) Leonard & Suggs] disease was investigated at the wax ripeness stage. Twenty plants were evaluated at each sampling point. Six disease scales were rated based on the percentage of the total leaf area affected: 0, no disease; 1, few lesion below the ear leaf covering less than 5% of leaf surface; 3, lesion below the ear leaf cover between 6 and 10% of leaf surface, few lesion above the ear leaf; 5, lesion below the ear leaf cover 11–30% of leaf surface, a few lesion above ear leaf; 7, lesion area below the ear leaf cover 31–70% of leaf surface, lots of lesion above the ear leaf; 9, large coalesced lesions, covering more than 70% of the leaf surface, and foliage completely destroyed.

Wheat stripe rust (*Puccinia striiformis* West. f. sp. *tritici* Erikss) disease severity was surveyed at milk-ripe stage. 100 flag leaves were evaluated at each sampling point. Disease severity were rated according to a linear scale of percentage of symptomatic leaf area from 0, 1%, 5%, 10%, 20%, 40%, 60%, 80% and 100%.

Potato late blight [*Phytophthora infestans* (Mont.) de Bary] disease was assessed at twenty days after potato flowering. Twenty plants were randomly selected to evaluate at each sampling point. Six disease scales were rated according to the percentage of leaflets area affected: 0, none lesions; 1, less than 5%; 3, more than 6% but less than 10%; 5, more than 11% but less than 20%; 7, more than 20% but less 50%, 9, more than 50% leaf area affected.

Sugarcane eye spot [*Bipolaris sacchari* (Butler) Shoemaker] disease was surveyed at the late stage of stalk elongation. Thirty plants were surveyed at each sampling point, Six disease scales was scored: 0, no disease; 1, few small brown spot on leaf; 2, lesion area about 3 mm×1 mm and the number less than 10; 3, lesion area about 4 mm×1.5 mm and the number more than 10; 4, lesion area about 5∼8 mm×1.5∼2.0 mm and the number more than 20, some lesions coalesced; 5, lesion area about 5∼8 mm×1.5∼2.0 mm and the number more than 30, some lesions coalesced and leaf destroyed.

Broad bean chocolate spot (*Botrytis fabae* Sard.) disease was surveyed on pod-setting stage. Thirty plants were randomly selected to evaluate at each sampling point. Five disease scales was scored: 0, no lesions or few small brown, non-sporulating specks, covering up to l% of leaf surface; 1, few small, discrete, brown, circular, nonsporulating lesions (2–3 mm in diameter) covering between 1.1 and 2% of leaf surface.; 2 is lesions common (3–5 mm in diameter) some coalesced, covering 2.1–5% of leaf surface, with some defoliation and very poor sporulating.; 3 is large coalesced irregular lesions which are blackish, sporulating, and cover 5.1–10% of leaf surface, average defoliation, flower drop, and some dead plants.; 4 is extensive large coalesced irregular lesions which are blackish, heavily sporulating, and cover more than 10% of the leaf surface, severe defoliation, stem girdling, and death of great majority of plants.

Disease severity was summarized within each plot as {[(n_1_×1)+(n_2_×2)+(n_3_×3)+…+(n_N_×N)]/N×(n_1_+n_2_+n_3_+…+n_N_)}×100, where n_1_… n_N_ is the number of leaves in each of the respective disease categories, N is the highest scoring of the disease; m = disease severity for crop species grown in monoculture control plots; i = disease severity for the same crop species grown in intercropping plots in the same fields. Error bars are one s.e.m; n = 3. Statistical analyses were conducted by software SPSS 13.0. All differences between pairs are significant at P≤0.05 based on one-tailed t-test.
